# Episodic Ethanol Exposure in Adolescent Rats Causes Residual Alterations in Endogenous Opioid Peptides

**DOI:** 10.3389/fpsyt.2018.00425

**Published:** 2018-09-10

**Authors:** Linnea Granholm, Lova Segerström, Ingrid Nylander

**Affiliations:** Department of Pharmaceutical Bioscience, Neuropharmacology, Addiction and Behaviour, Uppsala University, Uppsala, Sweden

**Keywords:** beta-endorphin, dynorphin B, enkephalin, rat model, developing brain, alcohol

## Abstract

Adolescent binge drinking is associated with an increased risk of substance use disorder, but how ethanol affects the central levels of endogenous opioid peptides is still not thoroughly investigated. The aim of this study was to examine the effect of repeated episodic ethanol exposure during adolescence on the tissue levels of three different endogenous opioid peptides in rats. Outbred Wistar rats received orogastric (i.e., gavage) ethanol for three consecutive days per week between 4 and 9 weeks of age. At 2 h and 3 weeks, respectively, after the last exposure, beta-endorphin, dynorphin B and Met-enkephalin-Arg^6^Phe^7^ (MEAP) were analyzed with radioimmunoassay. Beta-endorphin levels were low in the nucleus accumbens during ethanol intoxication. Remaining effects of adolescent ethanol exposure were found especially for MEAP, with low levels in the amygdala, and high in the substantia nigra and ventral tegmental area three weeks after the last exposure. In the hypothalamus and pituitary, the effects of ethanol on beta-endorphin were dependent on time from the last exposure. An interaction effect was also found in the accumbal levels of MEAP and nigral dynorphin B. These results demonstrate that repeated episodic exposure to ethanol during adolescence affected opioid peptide levels in regions involved in reward and reinforcement as well as stress response. These alterations in opioid networks after adolescent ethanol exposure could explain, in part, the increased risk for high ethanol consumption later in life.

## Introduction

During adolescence, social interactions with peers become highly important and increased frequencies in behaviors like risk-taking, impulsivity and novelty-seeking can be observed in experimental models (1). In the western world, many adolescents begin experimentation with ethanol during this period of life (2, 3). Exposure to ethanol may pose risks as indicated by findings showing that early onset of drug consumption can increase later susceptibility for drug abuse and addiction (4–7). This vulnerability could be a result of three factors (8). Firstly, adolescents frequent environments in which drugs are used. Secondly, early use could be a consequence of an inherited vulnerability for drugs of abuse. Thirdly, as the adolescent brain continually matures, early use might shape the brain toward a vulnerability state, which consequently leads to later susceptibility for drug use. To investigate the third factor, an adolescent rat model was used to study the endogenous opioid system after episodic binges of ethanol.

Ethanol has an unspecific mechanism of action as it does not have a specific target protein. Instead, ethanol acts on several receptors and ion channels in a number of transmitter networks, including the endogenous opioid system (9, 10). The endogenous opioids regulate other neurotransmitters of importance for reward and reinforcement (e.g., dopamine and γ-aminobutyric acid). Most drugs of abuse affect the endogenous opioid system and their effects on different brain target areas differ depending on the drug and also on the phase in the addiction cycle, i.e., binge/intoxication, withdrawal/negative affect, preoccupation/anticipation (9, 11, 12). The classical endogenous opioid system consists of three G-protein coupled receptors (μ-, δ-, and κ-receptors) and their corresponding ligands (endorphins, enkephalins and dynorphins). The endogenous opioid peptides are derived from precursors i.e., prohormones (13, 14). Beta-endorphin that binds to μ-receptors is generated from proopiomelanocortin (15). Dynorphin B is cleaved from prodynorphin (16) and binds to κ-receptors. Met-enkephalin-Arg^6^-Phe^7^ (MEAP) is derived from proenkephalin (17) and binds predominantly to δ-receptors but also to μ-receptors (18).

**Figure 1:**
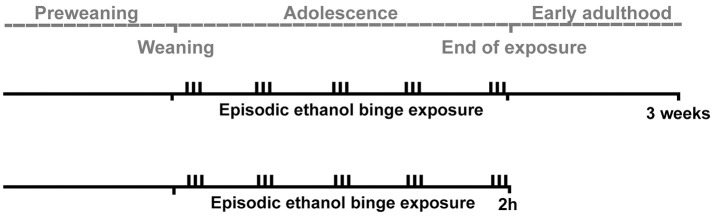
A schematic overview of the experiment. Adolescent male Wistar rats were exposed to episodic binges of ethanol three times per week (indicated by vertical bars) during adolescence. Two hours (in the intoxicated state) or three weeks (to measure residual effects) after the last exposure of ethanol, three endogenous opioids (beta-endorphin, dynorphin B and Met-Enkephalin-Arg^6^-Phe^7^) were measured in several brain areas. The gray dotted line indicates the timeline of the experiment.

This study used adolescent male Wistar rats to evaluate the effects of episodic binge-like exposure of ethanol on brain levels of the following three opioid peptides; beta-endorphin, dynorphin B, and MEAP. Levels were measured at 2 h, to investigate the effects of intoxication, and at 3 weeks, to study long-term, residual changes (Figure 1).

## Methods

### Animals and experimental design

All animal experiments were performed with the approval of the Uppsala Animal Ethical Committee and according to the principles of the Guide for the Care and Use of Laboratory Animals, the guidelines of the Swedish Legislation on Animal Experimentation (Animal Welfare Act SFS1998:56), and the EU Parliament and the Council Directive of 22 September 2010 (2010/63/EU).

Two sets of time-mated Wistar rats (Harlan Laboratories B.V., Horst, the Netherlands) arrived at the animal facility in Uppsala, Sweden, at gestation day 15. The dams were housed individually in a standard cage (59 × 38 × 20 cm) with wood chip bedding and nesting material under standard conditions (22°C, 50 ± 10% humidity, 12 h light-dark cycle commencing at 07:00, *ad libitum* access to pellet food and tap water, and background noise masking). The pregnant females were transported during the least sensitive phase of the gestation. No signs of negative impact of the travel were noticed and the delivery was normal in all females. To avoid biological littermates, the litters were cross-fostered and mixed on the day of birth (postnatal day, PND, 0) so each litter contained four females and six males. Previous studies have shown that single housing affects brain levels of endogenous opioid peptides in adolescent rats (19, 20), so on PND 21 the pups were weaned and group housed (2–3 rats per cage) to avoid confounding factors.

### Adolescent ethanol exposure

Between 4 and 9 weeks of age, the male rats received orogastric administration of water (*n* = 20) or ethanol, 2 g/kg, 20% v/v ethanol diluted with tap water, (*n* = 20).

Administrations were given at 09:00 on three consecutive days, followed by 4 days without treatment. Orogastric administration was used since it does not require single housing and this route of administration resemble the oral ingestion of ethanol by humans. Unpublished data from our pilot study and published data from others (21) have shown that 2 g/kg produces blood alcohol concentration reaching the National Institute on Alcohol Abuse and Alcoholism criterion for binge drinking (i.e., >0.08 g/dl in 2 h). The rats were housed under standard conditions as described above except that the light and dark cycles were reversed at weaning. The rats were euthanized by decapitation either 2 h or 3 weeks after the last ethanol exposure.

### Tissue stabilization and sampling

The pituitary glands was snap frozen on dry ice whereas the whole brains were immediately frozen in an isopentane bath (−20°C for 2 min). The tissues were stored at −80°C. One day prior to stabilization, the whole brains and the pituitaries were moved to a −20°C freezer to reduce the temperature gradient before stabilization. The tissue samples were stabilized by heat denaturation (95°C) with a bench-top Stabilizor T1 (Denator AB, Uppsala, Sweden) according to the manufacturer's manual. The stabilization process involves a combination of conductive heat transfer and pressure under vacuum to prevent enzymatic degradation (e.g., of peptides) during freeze-thawing (22). Whole brains were placed in a Maintainor Tissue card (Denator AB, Uppsala, Sweden) and stabilized in the “frozen structure preserve mode” and thereafter in the “fresh structure preserve mode” to ensure an adequate treatment. After stabilization, the brains were dissected according to Paxinos and Watson (23) to separate the hypothalamus, medial prefrontal cortex, cingulate cortex, dorsal striatum, nucleus accumbens, amygdala, hippocampus, ventral tegmental area (VTA) and substantia nigra. The pituitaries were individually placed in a pre-chilled Maintainor Tissue card and stabilized in the “frozen quick compress mode”. Stabilized tissues were thereafter stored at −80°C.

### Peptide extraction

The tissues were moved from −80°C and heated in 95°C acetic acid (1M) for 5 min, then placed on ice and homogenized by sonication using a Branson Sonifier (Danbury, CT, USA). The homogenates were centrifuged for 15 min at 4°C, 12,000 × *g* in a Beckman GS-15R centrifuge (Fullerton, CA, USA) and supernatants were purified by cation exchange chromatography procedure (24). The purified samples were dried in a vacuum centrifuge and stored at −20°C.

### Radioimmunoassay

Measurement of the immunoreactive levels of dynorphin B and MEAP was performed according to Nylander et al. (25, 26) with antisera generated in rabbits. The dynorphin B antiserum (113+) was used at a final dilution of 1:500,000. The cross-reactivity with DYNB 29 is 1% and with big dynorphin (DYN 32) 100%, whereas no other opioid peptide cross-reacts in the assay. The detection range in the dynorphin B assay is 1–70 fmol in 25 μl of the sample. The MEAP antiserum (90:3D II) was used at a final dilution of 1:140,000. The cross-reactivity with Met-enkephalin, Met-enkephalin-Arg^6^, Met-enkephalin-Arg^6^Gly^7^Leu^8^, Leu- enkephalin and dynorphin A (1–6) is less than 0.1% and no other opioid peptide cross-reacts in the assay. The detection range in the MEAP assay is 2-100 fmol in 25 μl of the sample. Antibody-bound peptides in the dynorphin B assay were separated from free peptides by adding goat-anti-rabbit-IgG and normal rabbit serum. For the MEAP assay, separation was performed by adding charcoal suspension (Sigma-Aldrich, MO, USA).

For the beta-endorphin, a commercial kit was used according to the manufacturer's instructions (Phoenix Pharmaceutical, Inc., Burlingame, CA, USA). Cross-reactivity was reported to be 100% with alpha-endorphin, 40% with human beta-endorphin but none with alpha-MSH, ACTH, PACAP 38, Met- or Leu-enkephalin and the detection range was 1–128 pg in 100 μl of the sample.

### Statistics

One-way analysis of variance (ANOVA) was used to investigate overall differences between the groups and effect size was estimated with the partial eta-squared test. Factorial ANOVAs were used to test the effects of treatment (adolescent exposure to ethanol or water), time (2 h or 3 weeks after the last exposure) or interaction (time × treatment). The factor time also represents a factor of age since the rats were 9 or 12 weeks of age at the time-point for decapitation, i.e., 2 h or 3 weeks after the last exposure. Significant levels were set to *p* < 0.05; Tukey's *post hoc* test was used to analyze between-group differences. Extreme values (1.5 standard deviation) within each treatment group were excluded from the analyses.

## Results

The statistical results for beta-endorphin, dynorphin B and MEAP in all brain tissues and the pituitary are given in Tables 1–3 respectively.

**Table 1:** Immunoreactive levels (fmol/mg) of beta-endorphin.

	**Water intoxication**	**Ethanol intoxication**	**Water residual effects**	**Ethanol residual effects**	**Two-factor ANOVA**			**η${}_{\text{p}}^{2}$**
	**Mean** ±**SEM**	**Mean** ±**SEM**	**Mean** ±**SEM**	**Mean** ±**SEM**	**Treatment**	**Time**	**Treatment** × **Time**	
Pít	*27.982*±*1.653*	*33.940*±*2.890*	*31.685*±*2.783*	*25.800*±*2.054*	*F*_(1, 35)_ < 0.01; *p* = 0.99	F_(1, 35)_ = 0.84; *p* = 0.37	***F***_(1, 35)_ = **5.99;** ***p*** = **0.020**	0.17
Ht	41.0 ± 1.3	44.4 ± 1.6	45.0 ± 3.7	37.5 ± 2.2	*F*_(1, 35)_ = 0.69; *p* = 0.41	*F*_(1, 35)_ = 0.35; *p* = 0.56	***F***_(1, 35)_ = **5.16;** ***p*** = **0.029**	0.15
AMY	3.5 ± 0.3	2.9 ± 0.2	3.1 ± 0.4	2.8 ± 0.2	*F*_(1, 35)_ = 2.84; *p* = 0.10	*F*_(1, 35)_ = 0.79; *p* = 0.38	*F*_(1, 35)_ = 0.36; *p* = 0.55	0.10
NAc	2.2^*^ ± 0.5	1.0^#^ ± 0.1	0.9 ± 0.1	1.1 ± 0.2	*F*_(1, 35)_ = 3.23; *p* = 0.081	***F***_(1, 35)_ = **4.89;** ***p*** = **0.034**	***F***_(1, 35)_ = **5.32;** ***p*** = **0.027**	0.28
VTA	2.9 ± 0.3	2.6 ± 0.2	2.7 ± 0.3	2.8 ± 0.3	*F*_(1, 34)_ = 0.14; *p* = 0.71	*F*_(1, 34)_ < 0.01; *p* = 0.99	*F*_(1, 34)_ = 0.37; *p* = 0.55	0.016
dStr	0.6 ± 0.1	0.4 ± 0.04	0.4 ± 0.04	0.4 ± 0.02	*F*_(1, 34)_ = 3.87; *p* = 0.057	***F***_(1, 34)_ = **5.84;** ***p*** = **0.021**	*F*_(1, 34)_ = 1.55; *p* = 0.22	0.25

Rats were repeatedly exposed to ethanol or water during adolescence. Two hours (during ethanol intoxication) or three weeks (residual effects) after the last exposure, the immunoreactive levels of beta-endorphin were measured in different brain regions. Amy, amygdala; ANOVA, analysis of variance; Ht, hypothalamus; NAc, nucleus accumbens; $\eta_{\text{p}}^{2}$, partial eta-squared; Pit, pituitary; Str, striatum; VTA, ventral tegmental area. Tukey's post hoc test;

* p < 0.05 intoxication effects (2 h) compared to the residual effects (3 weeks) of the same treatment;

# *p < 0.05 ethanol compared to water at the same time-point. Bold letters highlights statistically significant results*.

### Beta-endorphin

**Figure 2:**
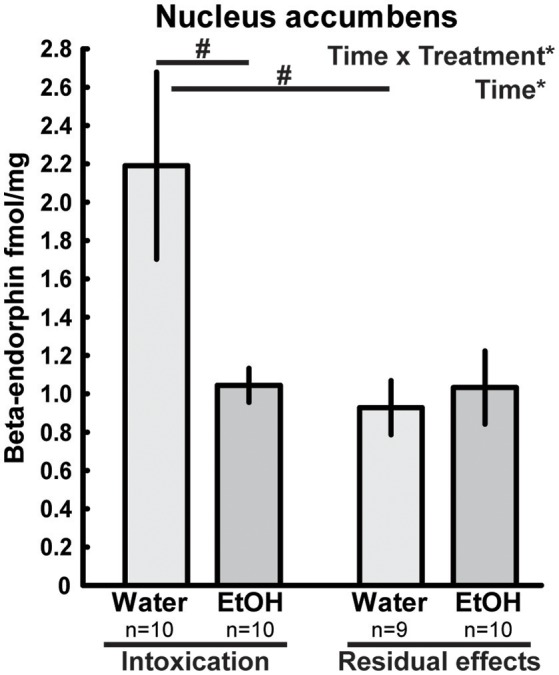
Beta-endorphin (fmol/mg tissue) in the nucleus accumbens after repeated adolescent ethanol (EtOH) exposure. Levels were measured in an ethanol-intoxicated state (2 h after last exposure) and 3 weeks after the exposure (residual effects). Data expressed as mean ± SEM. “Treatment × time” and “Treatment” indicates a significant interaction effect and an effect of treatment, respectively, *p < 0.05 two-factor ANOVA. ^#^*p* < 0.05 Tukey's HSD *post hoc* test.

In the nucleus accumbens, differences in beta-endorphin levels between the ethanol-treated rats and water controls were indicated by an interaction between time and treatment [*F*_(1, 35)_ = 5.32; *p* = 0.03]. Beta-endorphin levels were lower in the intoxicated state (i.e., after 2 h) than for their time-matched water controls; this effect was not present 3 weeks after the last exposure of ethanol (Figure 2).

**Figure 3:**
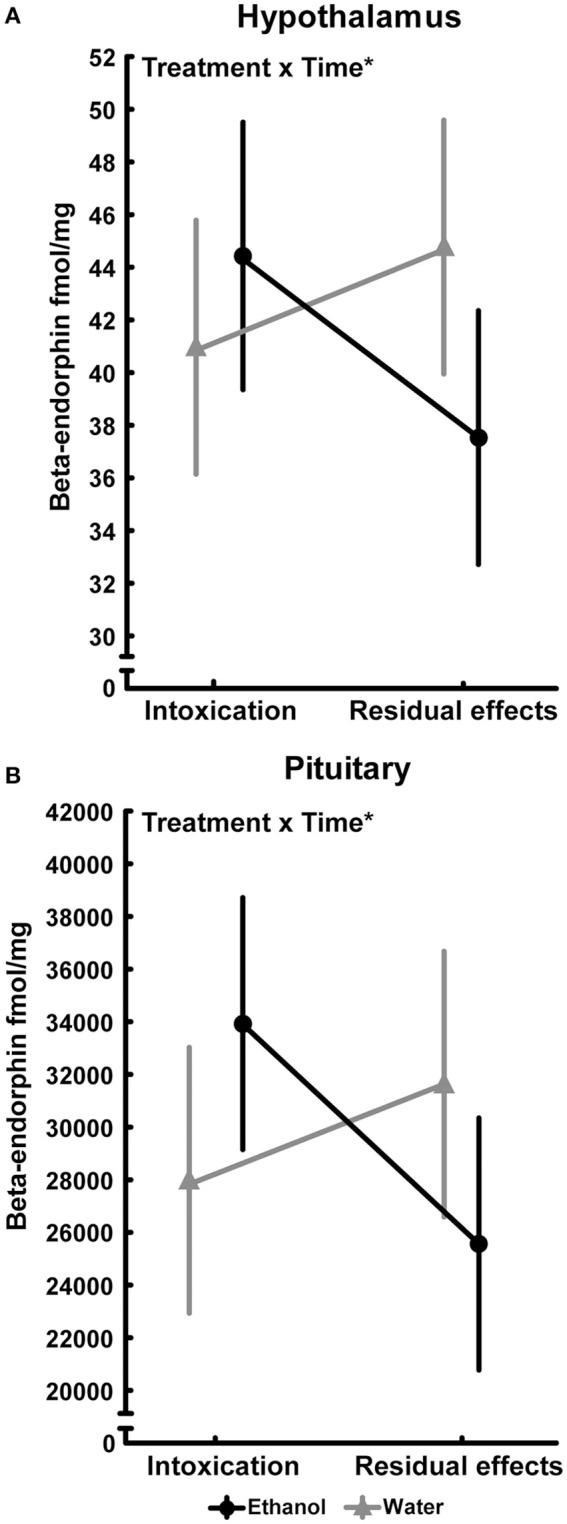
Beta-endorphin (fmol/mg tissue) in **(A)** hypothalamus (Intoxication; water, *n* = 10; ethanol, *n* = 9 and residual effects; water, *n* = 10; ethanol, *n* = 10) and **(B)** pituitary (Intoxication; water, *n* = 9; ethanol *n* = 10 and Residual effects; water, *n* = 9; ethanol, *n* = 10) after repeated adolescent ethanol exposure. Levels were measured in an ethanol-intoxicated state (2 h after last exposure) and 3 weeks after the last exposure (residual effects). Data expressed as mean ± SEM. “Treatment × time” indicates a significant inter-action effect (^*^p < 0.05 two-factor ANOVA).

An effect of time [*F*_(1, 35)_ = 4.89; *p* = 0.03] was also found in the nucleus accumbens and was driven by the higher beta-endorphin in the water group at 2 h. Interactions between treatment and time were also found in the hypothalamus [*F*_(1, 35)_ = 5.16; *p* = 0.03] and in pituitary [*F*_(1, 35)_ = 5.99; *p* = 0.02] but the Tukey's *post-hoc* test did not reveal any between-group differences, see Figure 3. An overall effect between the treatment groups was found in the dorsal striatum [*F*_(3, 34)_ = 3.85; *p* = 0.03], but *post hoc* analyses failed to identify any statistical between-group differences in beta-endorphin. Furthermore, the two-way ANOVA analysis showed an effect of time [F_(1, 34)_ = 5.84; *p* = 0.02] in the dorsal striatum (Table 1).

### Dynorphin B

**Figure 4:**
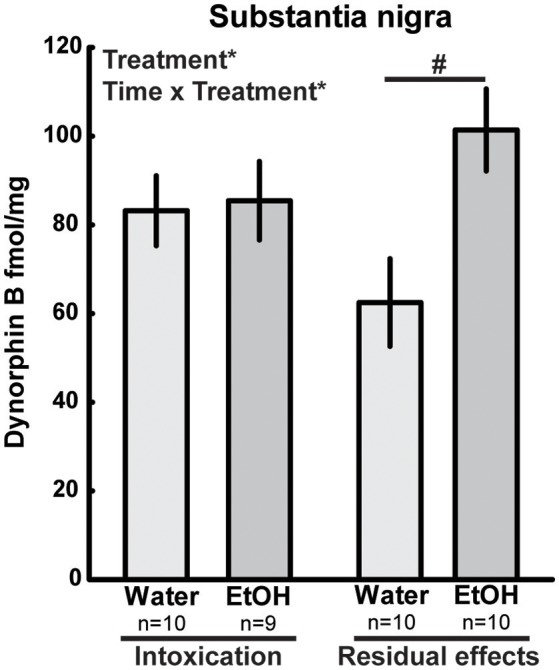
Dynorphin B (fmol/mg tissue) in substantia nigra after repeated adolescent ethanol (EtOH) exposure. Levels were measured in an ethanol-intoxicated state (2 h after last exposure) and 3 weeks after the last exposure (residual effects). Data expressed as mean ± SEM. “Treatment × time” and “Treatment” indicates a significant interaction effect and an effect of treatment, respectively, ^*^p < 0.05 two-factor ANOVA. ^#^*p* < 0.05 Tukey's HSD *post hoc* test.

In the pituitary, there was an overall effect of treatment [*F*_(1, 34)_ = 8.09; *p* = 0.01] and time [*F*_(1, 34)_ = 4.54; *p* = 0.04], and a trend (*p* = 0.06) of increased dynorphin B was seen in ethanol-intoxicated rats (Table 2). In the substantia nigra, there was an effect of treatment [*F*_(1, 35)_ = 5.21; *p* = 0.03] as well as an interaction effect [*F*_(1, 35)_ = 4.13; *p* = 0.05]. In the intoxicated state (2 h), there was no difference between the ethanol-treated group and water controls, but higher dynorphin B were found in the substantia nigra of the ethanol treated group at 3 weeks (Figure 4). For dynorphin B, an effect of time was present in the hypothalamus [*F*_(1, 36)_ = 4.76; *p* = 0.04], the nucleus accumbens [*F*_(1, 35)_ = 5.49; *p* = 0.02] and the hippocampus [*F*_(1, 36)_ = 4.76; *p* = 0.04] (Table 2).

**Table 2:** Immunoreactive levels (fmol/mg) of dynorphin B.

	**Water intoxication**	**Ethanol intoxication**	**Water residual effects**	**Ethanolresidual effects**	**Two-factor ANOVA**			**η${}_{\text{p}}^{2}$**
	**Mean** ±**SEM**	**Mean** ±**SEM**	**Mean** ±**SEM**	**Mean** ±**SEM**	**Treatment**	**Time**	**Treatment** × **Time**	
Pit	570.1^°^± 23.1	703.4 ± 32.6	677.8 ± 30.3	748.4 ± 48.7	***F***_(1, 34)_ = **8.09;** ***p*** = **0.01**	***F***_(1, 34)_ = **4.54;** ***p*** = **0.04**	*F*_(1, 34)_ = 0.76; *p* = 0.39	0.28
Ht	22.6 ± 2.3	21.8 ± 1.6	28.3 ± 1.4	29.2 ± 1.9	*F*_(1, 36)_ = 0.02; *p* = 0.88	***F***_(1, 36)_ = **4.76;** ***p*** = **0.04**	*F*_(1, 36)_ = 0.25; *p* = 0.62	0.20
AMY	9.8 ± 1.0	9.2 ± 0.8	8.9 ± 1.1	10.4 ± 0.8	*F*_(1, 35)_ = 0.22; *p* = 0.65	*F*_(1, 35)_ = 0.03; *p* = 0.87	*F*_(1, 35)_ = 1.23; *p* = 0.27	0.040
NAc	36.7^*^± 2.4	30.0 ± 1.6	28.7 ± 1.1	29.0 ± 2.2	*F*_(1, 35)_ = 2.73; *p* = 0.11	***F***_(1, 35)_ = **5.49;** ***p*** = **0.02**	*F*_(1, 35)_ = 3.24; *p* = 0.08	0.25
VTA	5.3 ± 0.6	5.7 ± 0.8	6.2 ± 1.2	8.3 ± 1.5	*F*_(1, 34)_ = 1.24; *p* = 0.27	*F*_(1, 34)_ = 2.62; *p* = 0.11	*F*_(1, 34)_ = 0.54; *p* = 0.47	0.12
SN	83.2 ± 7.9	85.4 ± 8.9	62.5 ± 9.9	101.4^#^±9.3	***F***_(1, 35)_ = **5.21;** ***p*** = **0.03**	*F*_(1, 35)_ = 0.07; *p* = 0.79	***F***_(1, 35)_ = **4.13;** ***p*** = **0.05**	0.22
dStr	16.1 ± 0.8	14.7 ± 1.5	15.5 ± 0.9	13.4 ± 0.6	*F*_(1, 35)_ = 2.91; *p* = 0.10	*F*_(1, 35)_ = 0.86; *p* = 0.36	*F*_(1, 35)_ = 0.13; *p* = 0.72	0.097
Hc	22.6 ± 2.3	21.8 ± 1.6	26.6 ± 2.7	28.1 ± 2.7	*F*_(1, 36)_ = 0.02; *p* = 0.88	***F***_(1, 36)_ = **4.76;** ***p*** = **0.04**	*F*_(1, 36)_ = 0.25; *p* = 0.62	0.12
CCx	1.4 ± 0.3	1.0 ± 0.2	1.2 ± 0.2	1.5 ± 0.3	*F*_(1, 35)_ = 0.02; *p* = 0.90	*F*_(1, 35)_ = 0.23; *p* = 0.64	*F*_(1, 35)_ = 1.83; *p* = 0.18	0.055
MPFCx	1.3 ± 0.1	1.1 ± 0.1	1.1 ± 0.2	1.2 ± 0.2	*F*_(1, 36)_ = 0.14; *p* = 0.71	*F*_(1, 36)_ = 0.06; *p* = 0.82	*F*_(1, 36)_ = 0.42; *p* = 0.52	0.017

Rats were repeatedly exposed to ethanol or water during adolescence. Two hours (during ethanol intoxication) or three weeks (residual effects) after the last exposure, the immunoreactive levels of dynorphin B were measured in different brain regions. Amy, amygdala; CCx, cingulate cortex; Hc, hippocampus; Ht, hypothalamus; DynB; mPFCx, medial prefrontal cortex; NAc, nucleus accumbens; $\eta_{\text{p}}^{2}$, partial eta squared; Pit, pituitary; SN, substantia nigra; Str, striatum; VTA, ventral tegmental area. Tukeys post hoc test;

* p < 0.05 intoxication effects (2 h) compared to the residual effects (3 weeks) of the same treatment;

# p < 0.05 ethanol compared to water at the same time-point;

° *p = 0.06 ethanol compared to water at the same time-point. Bold letters highlights statistically significant results*.

### Met-enkephalin-Arg^6^-Phe^7^

**Figure 5:**
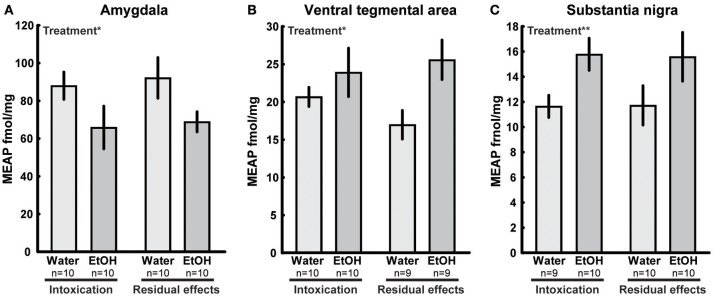
Met-Enkephalin-Arg^6^-Phe^7^ (MEAP) (fmol/mg tissue) in **(A)** amygdala, **(B)** ventral tegmental area, and **(C)** substantia nigra after repeated adolescent ethanol (EtOH) exposure. Levels were measured in an ethanol-intoxicated state (2 h after last exposure) and 3 weeks after the last exposure (residual effects). Data expressed as mean ± SEM. “Treatment” indicates a significant interaction effect of treatment, ^*^p < 0.05, ^**^p < 0.01 two-factor ANOVA.

**Figure 6:**
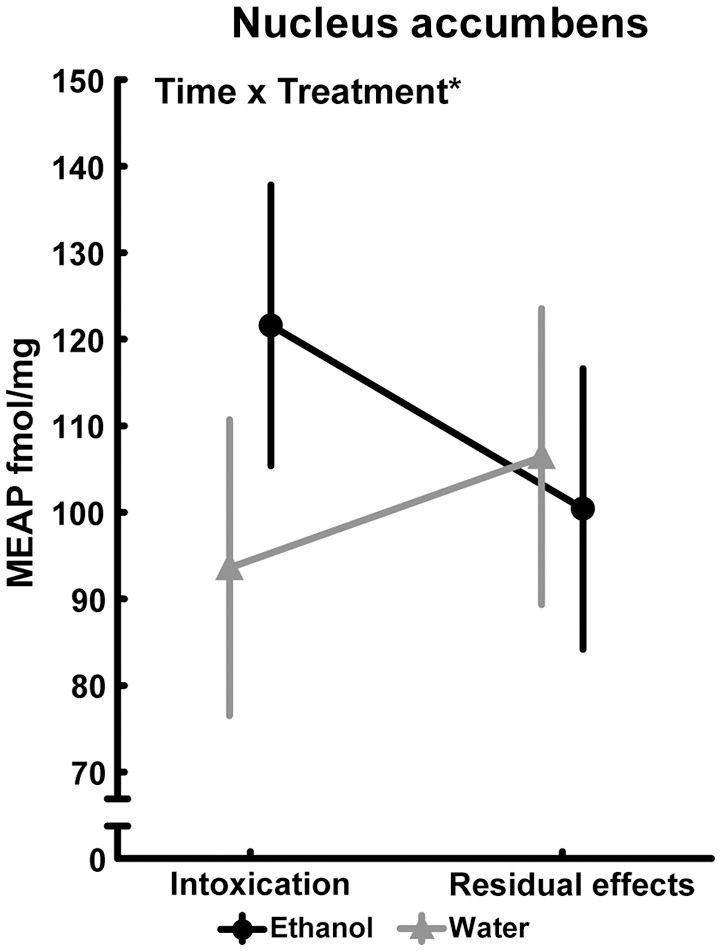
Met-Enkephalin-Arg^6^-Phe^7^ (MEAP) (fmol/mg tissue) in the nucleus accumbens after repeated adolescent ethanol exposure. Levels were measured in an ethanol-intoxicated state (2 h after last exposure) and 3 weeks after the last exposure (residual effects). Data expressed as mean ± SEM. “Treatment × time” indicates a significant interaction effect, ^*^p < 0.05 two-factor ANOVA. Intoxication; water, *n* = 10; ethanol, *n* = 10 and residual effects; water, *n* = 9; ethanol, *n* = 10.

In several brain areas, the effects of ethanol exposure on MEAP levels persisted 3 weeks after the last exposure to ethanol. In the amygdala, an effect of treatment [*F*_(1, 36)_ = 6.33; *p* = 0.02] was found with lower MEAP after ethanol exposure (Figure 5A). In the VTA [*F*_(1, 34)_ = 6.20; *p* = 0.02] and substantia nigra [*F*_(1, 35)_ = 7.15; *p* = 0.01], the levels were higher in ethanol-exposed rats (Figures 5B,C). In the above-mentioned structures, Tukey's *post hoc* test did not reveal any between-group differences. There was a significant overall effect [*F*_(3, 33)_ = 2.97; *p* = 0.05] in the pituitary, but *post hoc* analysis showed only a strong trend (*p* = 0.053) of higher MEAP in the ethanol-intoxicated rats (Table 3). In the nucleus accumbens, an interaction effect [*F*_(1, 34)_ = 4.31; *p* = 0.05] showed that the levels of MEAP varied, depending on both treatment and time but there was no significant differences between the groups (Figure 6). An effect of time, i.e., 2 h or 3 weeks after the last ethanol exposure, was found in the hypothalamus [*F*_(1, 35)_ = 17.07; *p* < 0.001]. MEAP levels were found to be lower at 3 weeks than at 2 h for both the ethanol and control groups (Table 3). The same effect of time was also present in the dorsal striatum [*F*_(1, 34)_ = 8.60; *p* = 0.006] (Table 3).

**Table 3:** Immunoreactive levels (fmol/mg) of Met-Enkephalin-Arg^6^-Phe^7^ (MEAP).

	**Water intoxication**	**Ethanol intoxication**	**Water residual effects**	**Ethanol residual effects**	**Two-factor ANOVA**			**η${}_{\text{p}}^{2}$**
	**Mean** ±**SEM**	**Mean** ±**SEM**	**Mean** ±**SEM**	**Mean** ±**SEM**	**Treatment**	**Time**	**Treatment x Time**	
Pit	12.6 ± 2.1	33.9^°^ ± 9.1	17.1 ± 3.1	17.6 ± 3.1	*F*_(1, 33)_ = 3.88; *p* = 0.06	*F*_(1, 33)_ = 1.15; *p* = 0.3	*F*_(1, 33)_ = 3.58; *p* = 0.07	0.12
Ht	129.2^*^ ± 4.4	125.3^*^ ± 6.5	107.1 ± 6.9	99.3 ± 5.2	*F*_(1, 35)_ = 0.98; *p* = 0.3	***F***_(1, 35)_ = **17.07;** ***p***<**0.001**	*F*_(1, 35)_ = 0.11; *p* = 0.8	0.34
AMY	88.0 ± 7.2	65.9 ± 11.3	92.2 ± 10.8	68.9 ± 5.3	***F***_(1, 36)_ = **6.33;** ***p*** = **0.02**	*F*_(1, 36)_ = 0.16; *p* = 0.7	*F*_(1, 36)_ = 0.0041; *p* = 0.9	0.15
NAc	93.6 ± 7.6	121.6 ± 6.8	106.4 ± 10.5	100.4 ± 7.7	*F*_(1, 34)_ = 1.79; *p* = 0.2	*F*_(1, 34)_ = 0.26; *p* = 0.6	***F***_(1, 34)_ = **4.31;** ***p*** = **0.05**	0.16
VTA	20.7 ± 1.3	23.9 ± 3.2	16.9 ± 1.9	25.6 ± 2.6	***F***_(1, 34)_ = **6.20;** ***p*** = **0.02**	*F*_(1, 34)_ = 0.18; *p* = 0.7	*F*_(1, 34)_ = 1.27; *p* = 0.3	0.18
SN	11.6 ± 0.9	15.8 ± 1.3	11.7 ± 1.6	15.6 ± 1.9	***F***_(1, 35)_ = **7.15;** ***p*** = **0.01**	*F*_(1, 35)_ = 0.0016; *p* = 0.9	*F*_(1, 35)_ = 0.0089; *p* = 0.9	0.16
dStr	85.1^*^ ± 5.8	81.5 ± 6.1	60.9 ± 4.9	71.5 ± 6.1	*F*_(1, 34)_ = 0.36; *p* = 0.6	***F***_(1, 34)_ = **8.60;** ***p*** = **0.006**	*F*_(1, 34)_ = 1.50; *p* = 0.2	0.23
Hc	9.6 ± 0.9	10.5 ± 0.7	9.4 ± 1.3	9.8 ± 1.2	*F*_(1, 36)_ = 0.35; *p* = 0.6	*F*_(1, 36)_ = 0.18; *p* = 0.7	*F*_(1, 36)_ = 0.069; *p* = 0.8	0.016
CCx	2.0 ± 0.4	1.8 ± 0.3	1.6 ± 0.3	2.3 ± 0.5	*F*_(1, 35)_ = 0.37; *p* = 0.5	*F*_(1, 35)_ = 0.014; *p* = 0.9	*F*_(1, 35)_ = 1.33; *p* = 0.3	0.048
MPFCx	5.8 ± 0.5	7.1 ± 0.6	7.0 ± 1.7	5.9 ± 0.4	*F*_(1, 36)_ = 0.023; *p* = 0.9	*F*_(1, 36)_ = 0.0014; *p* = 0.9	*F*_(1, 36)_ = 1.91; *p* = 0.2	0.043

Rats were exposed to ethanol or water during adolescence. Two hours (during ethanol intoxication) or three weeks (residual effects) after the last exposure, the immunoreactive levels of MEAP were measured in different brain regions. Amy, amygdala; CCx, cingulate cortex; Hc, hippocampus; Ht, hypothalamus; mPFCx, medial prefrontal cortex; NAc, nucleus accumbens; Pit, pituitary; SN, substantia nigra; Str, striatum; VTA, ventral tegmental area; w, weeks. Tukeys post hoc test;

* p < 0.05 intoxication effects (2 h) compared to the residual effects (3 weeks) of the same treatment;

° *p = 0.052 compared to water at the same time-point. Bold letters highlights statistically significant results*.

## Discussion

The neurobiological consequences of ethanol exposure during adolescence have been a neglected field in preclinical research until only recently [for recent reviews see (27, 28)]. There is, for example, still a knowledge gap in how ethanol affects the endogenous opioids in the adolescent brain, and the literature regarding the effect of repeated ethanol exposure during adolescence is almost nonexistent. To our knowledge, this is the first study to investigate the pharmacological effects of repeated adolescent ethanol exposure on the endogenous opioids, including both intoxication effects and residual effects 3 weeks after the exposure.

Previous studies from our laboratory have reported the effects of ethanol on endogenous opioids in adult rats as a function of strain, housing condition and ethanol administration paradigm (29–31). The differences for adult vs. adolescent rats must be compared carefully as they could be due to age, ethanol administration model, or both. In both adult and adolescent rats, the central levels of endogenous opioids interact with housing conditions (i.e., single or group housed) and ethanol intake (19, 20). These aforementioned studies show the profound importance of the experimental settings when working with ethanol models in rats. Therefore, to evaluate the pharmacological effects of ethanol exposure during adolescence, our rats were housed in groups and the ethanol was administered orogastrically by gavage to control the doses received. The present study focused on the effects in male rats and how ethanol affects the endogenous opioid peptides in females remains to be examined.

### Repeated ethanol exposure during adolescence and intoxication effects

In the present study, MEAP levels were increased in the pituitary 2 h after the last exposure. Palm and Nylander (20) presented similar results with increased MEAP 2 h after last drinking session in both single and group housed rats. This indicates a pharmacological effect of increased MEAP in the pituitary during intoxication. A trend (*p* = 0.06) toward increased dynorphin B was also found in the pituitary of the ethanol intoxicated rats. These changes may reflect ethanol involvement in stress axis activation as previously been reported [for review see Zhou and Kreek (32)].

Effects of intoxication were also found in nucleus accumbens where beta-endorphin was lower in the ethanol-exposed rats. This finding is in contrast to studies on adult rats, that report increased beta-endorphin in the nucleus accumbens after acute ethanol exposure (33, 34). This difference could be due to the choice of methodology, i.e., measuring peptide content in dialysate vs. tissue content, or it could be due to the effect of intoxication after just a single exposure vs. repeated exposure as in our study. The low beta-endorphin could be an indirect effect caused by ethanol-induced alterations in social behavior. Social play behavior activates the endogenous opioid system (35), specifically, the μ-receptors in the nucleus accumbens (36, 37). Ethanol has been shown to interfere with social play behavior; at low doses (0.25–0.75 g/kg) this behavior increase whereas at higher doses (1–4 g/kg) it decrease (38). In the present study, 2 g/kg ethanol was administered during the age interval (4–9 weeks of age) in which play behavior is reported to peak (39). Hence, the ethanol exposure could have affected the normal play behavior and thus social development, which could explain the differences in beta-endorphin levels in the nucleus accumbens. Another plausible explanation is that the high levels in water controls at the 2-h time-point is a consequence of stress-induced activation of beta-endorphin networks by the orogastric administration, considered a mild stressor (40), and that this effect is blunted by ethanol in the intoxicated animals. Differences between the ethanol-treated rats and water controls were not seen at the other time-point when 3 weeks had passed from the handling procedure. Changes in beta-endorphin were also seen as an interaction effect between time and treatment in the hypothalamus and pituitary, indicating possible effects of the handling procedure. Intermittent exposure of ethanol in adolescence have been reported to increase the expression of *pomc* in hypothalamus along with an increase of histone acetylation of the gene promotor (41).

An interaction between treatment (ethanol or water) and time (2 h or 3 weeks after the last exposure) was seen in the accumbal levels of MEAP, with the highest levels occurring in the intoxicated state. Previous studies have shown that ethanol intoxication increase enkephalins in the nucleus accumbens of adult rats. An increase of *Penk* expression and δ-receptor binding in shell and core of accumbens can be seen 2 h after ethanol administration (42, 43). Awake rats have increased accumbal levels of Met-enkephalin when injected with 1.6 g/kg ethanol, whereas higher (2.4 or 3.2 g/kg) or lower (0.8 g/kg) doses have no effect on Met-enkephalin (44). In anesthetized rats, the highest dose of ethanol (2.5 g/kg) leads to a peak of Met-enkephalin at 30 min, but lower doses (0.5 or 1.0 g/kg) delay the peak 90 and 60 min respectively (45). Furthermore, adolescent ethanol exposure alters the expression of *Penk* in the nucleus accumbens after an acute ethanol challenge in adult rats (46).

### Residual effects after repeated ethanol exposure during adolescence

An interesting finding was the residual effects of adolescent ethanol exposure on MEAP, such as the lower MEAP in the amygdala observed 3 weeks after the last exposure. The enkephalin system in amygdala is involved in emotional processing of states such as anxiety and stress (47) and *Oprd1* and *Penk* knock-out mice show increased anxiety and depressive like behaviors in a variety of tests (48–50). Pharmacological studies with systemic administrations and local injections of δ-receptor agonists into the amygdala decrease anxious behavior (51–53). Likewise, the administration of antagonists (51, 52, 54, 55) increases anxiety-like behaviors.

The scope herein was not to study behavioral manifestations *per se*, but the finding of residual low levels of enkephalin in the amygdala after adolescent exposure to ethanol indeed indicates long-lasting consequences that could relate to the increase in anxiety-like behaviors reported by others (41, 56, 57). δ-receptor knockout mice have an increased consumption of ethanol (58) and their elevated intake may be a way to reduce their elevated anxiety level (47). The low enkephalin tone after adolescent ethanol exposure may therefore constitute a risk factor for elevated intake of ethanol later in life. As noted above, an interaction between time and treatment was found in beta-endorphin in the hypothalamus and in the pituitary—these brain areas, along with the amygdala, are important in the regulation of the stress response.

Repeated exposure of ethanol during adolescence increased MEAP and dynorphin B in substantia nigra and MEAP in the VTA. The substantia nigra and VTA contain dopaminergic neurons that extend into the striatal, limbic and cortical areas (59). Importantly, endogenous opioids are highly involved in regulating dopamine output (60–62) and residual effects after adolescent ethanol exposure may have consequences for opioid regulation of dopamine pathways. Adolescent ethanol exposure has been shown to alter the dopamine dynamics in the dorsal striatum (63, 64), nucleus accumbens (65–67) and medial prefrontal cortex (68). An interesting aspect for future research would be to investigate the relationships between dopamine and opioid changes after adolescent ethanol exposure.

## Conclusion

Intoxication after repeated ethanol exposure during adolescence altered the levels of MEAP and beta-endorphin in the accumbens and dynorphin B and MEAP in the pituitary. Especially noteworthy is the observation of long-term consequences of the adolescent ethanol exposure, particularly MEAP in the amygdala and beta-endorphin in the hypothalamus and pituitary as these regions are involved in the response to anxiety and stress. Furthermore, residual effects were noted in the substantia nigra and VTA, areas important for opioid regulation of dopaminergic projections in the reward circuitry. It has been postulated that changes in stress circuits and in dopaminergic activity increase the susceptibility for alcohol use disorders. Hopefully, the data presented herein on the alterations in endogenous opioids after adolescent ethanol exposure can contribute in the understanding of how adolescent ethanol exposure increases the risk of elevated alcohol consumption later in life.

## Author contributions

IN, LG, and LS, experimental design. LG and LS, experimental work. LG, statistical analyses. LG, writing of the first draft. LS and IN, critical revision of the manuscript. LG, LS, and IN, finalization and approval of manuscript content.

### Conflict of interest statement

The authors declare that the research was conducted in the absence of any commercial or financial relationships that could be construed as a potential conflict of interest. The reviewer AO and handling Editor declared their shared affiliation at the time of the review

## Abbreviations

ANOVA: Analysis of variance
MEAP: Met-Enkephalin-Arg^6^-Phe^7^
VTA: Ventral tegmental area.
